# The Protein Quality Control Network in *Caulobacter crescentus*

**DOI:** 10.3389/fmolb.2021.682967

**Published:** 2021-04-30

**Authors:** Kristen Schroeder, Kristina Jonas

**Affiliations:** Science for Life Laboratory, Department of Molecular Biosciences, The Wenner-Gren Institute, Stockholm University, Stockholm, Sweden

**Keywords:** protease, chaperone, holdase, protein quality control, cell cycle, bacterial development

## Abstract

The asymmetric life cycle of *Caulobacter crescentus* has provided a model in which to study how protein quality control (PQC) networks interface with cell cycle and developmental processes, and how the functions of these systems change during exposure to stress. As in most bacteria, the PQC network of *Caulobacter* contains highly conserved ATP-dependent chaperones and proteases as well as more specialized holdases. During growth in optimal conditions, these systems support a regulated circuit of protein synthesis and degradation that drives cell differentiation and cell cycle progression. When stress conditions threaten the proteome, most components of the *Caulobacter* proteostasis network are upregulated and switch to survival functions that prevent, revert, and remove protein damage, while simultaneously pausing the cell cycle in order to regain protein homeostasis. The specialized physiology of *Caulobacter* influences how it copes with proteotoxic stress, such as in the global management of damaged proteins during recovery as well as in cell type-specific stress responses. Our mini-review highlights the discoveries that have been made in how *Caulobacter* utilizes its PQC network for regulating its life cycle under optimal and proteotoxic stress conditions, and discusses open research questions in this model.

## Introduction

**FIGURE 1:**
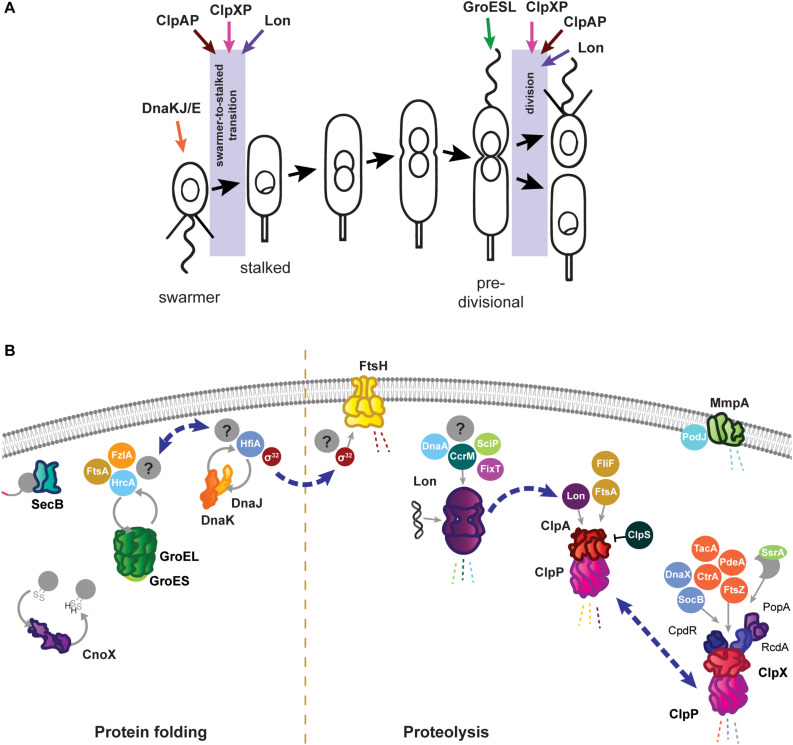
Roles of the *Caulobacter crescentus* PQC network in cell cycle progression and development. **(A)** The asymmetric life cycle of *Caulobacter*. Points where mechanisms have been identified where specific PQC proteins contribute to cell cycle progression are indicated with colored arrows. **(B)** Specific tasks of individual PQC network proteins in development and cell cycle progression during optimal conditions. Client proteins are indicated in circles where they are known, and by question marks where additional substrates have yet to be identified. Holdase cycling is indicated with gray circular arrows, and degradation with dashed lines. Blue dashed arrows indicate points of interaction between PQC network proteins. Membrane and DNA images created with Biorender (Biorender.com).

The aquatic alpha-proteobacterium *Caulobacter crescentus* (hereafter *Caulobacter*) is well-established as a model of bacterial cell cycle control and development, and is also used to study how prokaryotic protein quality control (PQC) networks interface with these processes. *Caulobacter* reproduces by an asymmetric life cycle, where division results in one replication-competent, surface-attached stalked cell and one chemotactically-motivated, non-replicative swarmer cell (Figure 1A; Curtis and Brun, 2010). The swarmer cell is motile and travels the environment until nutritional cues prompt its differentiation into a stalked cell, thus completing the cell cycle. To achieve this dimorphic lifestyle, processes from DNA replication to chemotaxis must be correctly organized in time and space. The synthesis and degradation of the proteins that implement these processes relies heavily on the PQC network during optimal growth conditions, but the PQC network must balance these tasks with the protective tasks required to survive stresses free-living bacteria frequently encounter. Initial work on the *Caulobacter* PQC network sought to identify if the bacterial PQC network performs a role in bacterial development (Gomes et al., 1986; Reuter and Shapiro, 1987). Since then, the major chaperones and proteases have been discovered to perform many regulatory functions in the *Caulobacter* cell cycle during optimal

conditions that are remodeled or modified during stress. These studies have collectively built a platform on which to address how a prokaryotic PQC network navigates the balance between reproductive and stress response tasks in order to mediate both growth and survival. The *Caulobacter* model is also used as a tool to answer questions of damage inheritance in asymmetric division, how generalist PQC networks can be specialized, and how stress responses are dynamically tailored.

As in other Gram negative bacteria, the primary energy-dependent nodes of the *Caulobacter* PQC network include the highly conserved chaperones and proteases GroES/EL, DnaKJ/GrpE, ClpB, ClpAP, ClpXP, Lon, FtsH, and HslUV (Figure 1B). The mechanism of action of these PQC machines are thought to be conserved among bacteria, and are reviewed in Balchin et al. (2016) and Mogk et al. (2018). *Caulobacter* additionally uses ATP-independent adaptor proteins, stress-specific holdases, inhibitory proteins, and specialized transcriptional regulation to further direct and specify the activities of its chaperones and proteases. In this mini-review we highlight the tasks of the *Caulobacter* PQC network that contribute to cell cycle progression and development during optimal conditions, and discuss how the nodes of this network reorganize during stress to perform protective tasks that are crucial for survival.

## Functions of the Energy-Dependent Folding Machines in Cell Cycle and Stress Adaptation

*Caulobacter* energy-dependent folding machines are capable of interacting broadly with the proteome to assist proteins into their native conformations, and perform specific and essential tasks both in cell cycle progression and stress response. For protein folding, *Caulobacter* utilizes the chaperone DnaK, co-chaperone DnaJ, and nucleotide exchange factor GrpE (DnaKJ/E), and a single copy of the chaperonin GroEL and co-chaperonin GroES (GroESL). In addition to DnaKJ/E, the *Caulobacter* genome contains one other DnaK-like protein (CCNA_01543) and five additional DnaJ-like proteins containing the characteristic J domain (CCNA_00965, CCNA_02218, CCNA_02245, CCNA_02860, CCNA_03105); however, it is currently unknown if these proteins direct the specificity of DnaK folding toward different client protein pools (Kampinga and Craig, 2010), or if they have another role. Depletion of either DnaKJ/E or GroESL halts the *Caulobacter* cell cycle in distinct stages; loss of DnaKJ/E results in a block of DNA replication initiation (Jonas et al., 2013; Schramm et al., 2017), whereas depletion of GroESL results in a cell division defect (Susin et al., 2006; Schroeder et al., 2020). Mild depletion of either of these folding machines produces an increase in the other (Da Silva et al., 2003; Susin et al., 2006), suggesting some degree of compensation exists, yet neither DnaKJ/E nor GroESL can fully substitute the stress response or cell cycle functions of the other.

The *Caulobacter* DnaKJ/E folding machine is essential in all growth temperatures, however, its function as a chaperone is dispensable in the absence of proteotoxic stress (Schramm et al., 2017). Instead, the requirement of DnaKJ/E for viability in optimal conditions is attributed to its binding and destabilization of the heat shock sigma factor σ^32^ (Da Silva et al., 2003; Schramm et al., 2017). In line with this notion, suppressor mutations reducing the abundance or activity of σ^32^ restore viability of cells depleted of DnaKJ in optimal conditions (Schramm et al., 2017). Sequestering of σ^32^ by DnaKJ/E prevents the sigma factor from inducing the expression of heat shock proteins (HSPs), which collectively function as a protective response that slows growth and is counterproductive in the absence of stress (Schramm et al., 2017). The importance of maintaining σ^32^ sequestration is reflected during depletion of DnaKJ/E in otherwise optimal conditions, where inappropriate HSP induction leads to a block in DNA replication through degradation of the replication initiator DnaA by the protease Lon (Jonas et al., 2013). DnaKJ/E also plays a role in *Caulobacter* development by interacting with the holdfast inhibitor HfiA (Eaton et al., 2016). DnaKJ/E activity keeps HfiA stabilized in a folded form, and this interaction operates in a regulatory circuit where increased levels of DnaK reduce the likelihood of developing surface attachment (Eaton et al., 2016), potentially promoting dispersal away from environments with inherent proteotoxic attributes.

**FIGURE 2:**
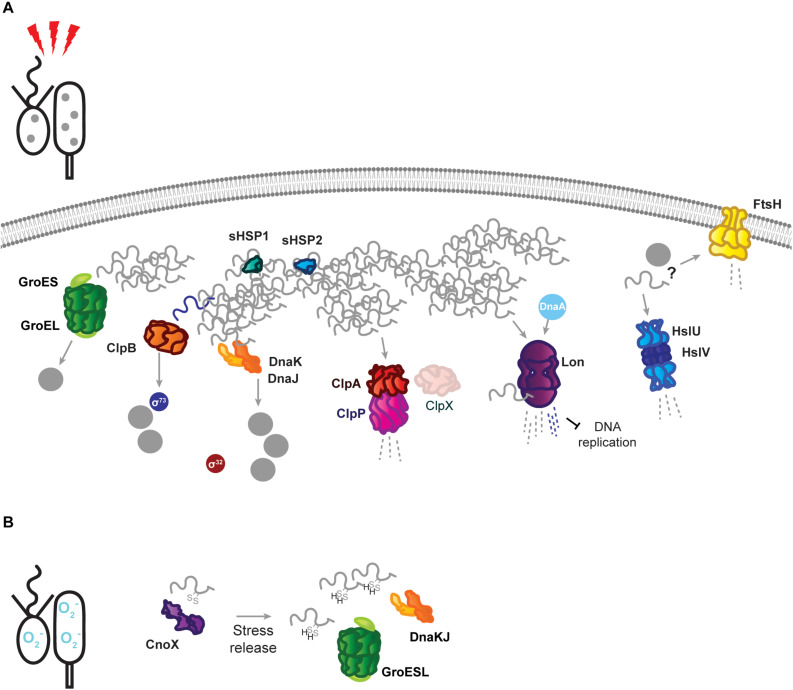
Stress response tasks of the *Caulobacter crescentus* PQC network. **(A)** Heat stress induces unfolding of the susceptible proteome, and unfolded proteins (gray squiggles) are incorporated into insoluble protein aggregates (gray dots). Known interactions during stress are indicated by protein name in colored circles. DnaKJ/E, ClpB, and GroESL participate in protein refolding and disaggregation. The small heat shock proteins organize unfolded proteins. ClpAP and Lon participate in degradation of unfolded proteins, in addition to regulatory roles. The proteases FtsH and HslUV are upregulated in response to proteotoxic stress, but it is unknown whether they contribute to degradation of unfolded proteins or regulatory substrates. **(B)** Oxidative stress results in oxidation of proteins and draining of the hydrotrope ATP, which can influence folding state. The holdase CnoX interacts and is capable of reducing disulfide groups of proteins, and protects them from aggregation until active GroESL and/or DnaKJ/E are available to refold these proteins. Membrane and DNA images created with Biorender (Biorender.com).

When proteotoxic stress conditions are encountered, DnaKJ/E is titrated away from σ^32^ by unfolded proteins, and here its folding activity becomes crucial to survival (Figure 2A). Under proteotoxic threat, liberation of σ^32^ results in induction of heat shock genes, including *dnaKJ* itself, which is expressed from a σ^32^-responsive promoter in addition to a constitutive (σ^73^-responsive) promoter (Avedissian et al., 1995; Reisenauer et al., 1996; Da Silva et al., 2003). The conditional switching of DnaKJ/E between its functions as a σ^32^ regulator and a folding catalyst is reflected by dynamic changes in its subcellular localization, as DnaKJ/E alternates between a dispersed pattern in optimal conditions and localization at foci of protein aggregation during stress (Schramm et al., 2019).

The chaperonin GroESL is expressed from a single promoter thought to respond to both σ^73^ and σ^32^ (Avedissian and Gomes, 1996; Baldini et al., 1998). During optimal conditions *Caulobacter groESL* is subject to a negative regulatory loop, effected through a controlling inverted repeat of chaperone expression (CIRCE) element and the HrcA repressor (Roberts et al., 1996; Baldini et al., 1998; Susin et al., 2004). Here, GroESL activity maintains HrcA in a folded conformation, in which it can bind the CIRCE element present in the *groESL* promoter, to reduce expression (Roberts et al., 1996; Baldini et al., 1998; Susin et al., 2004). Through CIRCE/HrcA regulation, the *groESL* transcript is cell cycle-regulated (Avedissian and Gomes, 1996; Baldini et al., 1998; Fang et al., 2013), and early pulse-chase experiments suggested that chaperonin synthesis is increased in the swarmer cell (Reuter and Shapiro, 1987). However, as GroESL protein is stably detected throughout the cell cycle in synchronized cultures (unpublished data), the relevance of this boost of synthesis remains unclear.

An overview of *Caulobacter* proteins whose folding state, or solubility, is influenced by GroESL has recently been described (Schroeder et al., 2020). Through this approach, cell cycle-regulated proteins involved in peptidoglycan biosynthesis and cell division were identified to have an interaction with GroESL folding availability in optimal conditions, including the FtsZ-interacting proteins FtsA and FzlA, which mediate a cell division block during GroESL depletion (Schroeder et al., 2020). While the role of GroESL in cell cycle progression is beginning to be understood, the contributions of this highly stress-induced folding machine during stress conditions have not yet been uncovered. In heat and ethanol stress σ^32^ induces robust *groESL* expression (Reuter and Shapiro, 1987; Susin et al., 2006; Heinrich et al., 2016), and while it is known that this *groESL* induction is specifically required to survive heat stress (Da Silva et al., 2003; Susin et al., 2006), the mechanisms by which GroESL protects the proteome during stress are not currently known.

## Proteases Are Integral to Driving Cell Cycle Progression and Are Tailored to Specific Stress Survival Tasks

Approximately 5% of proteins are estimated to be rapidly turned over during the *Caulobacter* cell cycle (Grünenfelder et al., 2001), and the use of proteolysis as a means of rapidly removing regulatory and structural proteins is fully integrated in remodeling the proteome during *Caulobacter* cell cycle progression. The regulatory networks and mechanisms by which proteolysis is integrated into the *Caulobacter* cell cycle have been discussed in detail in other recent reviews (Joshi and Chien, 2016; Vass et al., 2016). In addition to proteome curation and regulatory degradation during optimal conditions, *Caulobacter* proteases are stress-responsive and remove damaged proteins that accumulate during proteotoxic stresses, additionally functioning to halt the cell cycle and redirect available resources toward survival tasks.

The best-studied *Caulobacter* protease is ClpP, which can associate with either of the unfoldase subunits ClpX and ClpA (Figure 1B). ClpXP has many cell cycle-regulated targets, one of which is the master cell cycle regulator CtrA (Quon et al., 1996, 1998; Laub et al., 2002), and extensive work has uncovered that the regulated and coordinated activities of three specific adaptor proteins, CpdR, RcdA, and PopA, facilitate CtrA degradation at the correct time and location during the cell cycle (reviewed by Joshi and Chien, 2016). In addition to CtrA, several other proteins with critical functions in *Caulobacter* development are degraded by ClpXP, including PdeA (Abel et al., 2011), McpA (Tsai and Alley, 2001), TacA (Bhat et al., 2013), and FtsZ (Williams et al., 2014). Subsets of the ClpXP adaptors regulate degradation of several of these substrates (Joshi et al., 2015; Lau et al., 2015), emphasizing their importance in directing this protease toward specific substrate groups. While ClpX and ClpP are both essential in optimal conditions (Østerås et al., 1999), loss of ClpXP degradation of CtrA and other cell cycle substrates does not result in inviability. Instead, ClpXP degradation of the toxin SocB is essential to avoid inhibition of DNA polymerase activity, as suppressor mutations in *socB* bypass the need for ClpXP (Aakre et al., 2013). In addition to direct functions in cell cycle progression, ClpXP processing of the replication clamp subunit DnaX is required to promote DNA replication during optimal conditions, and accumulation of full length DnaX during genotoxic stress is an important factor in surviving DNA damage (Vass and Chien, 2013). Furthermore, ClpXP maintains a conserved function in degrading incompletely synthesized proteins directed from the SsrA/SspB pathway in *Caulobacter* (Keiler et al., 2000). Interestingly, the SsrA RNA (also known as tmRNA) of this system, which adds a degradation tag to products of stalled translation, is involved in cell cycle regulation, as deletion of *ssrA* delays timing of *dnaA* transcription and DNA replication (Keiler and Shapiro, 2003; Cheng and Keiler, 2009), however the precise mechanism remains unclear.

Unlike most other PQC network proteins, *clpX* expression is not induced by σ^32^ (Østerås et al., 1999; Schramm et al., 2017), however, both *clpA* and *clpP* expression are strongly upregulated by σ^32^ (Schramm et al., 2017). To accomplish this regulation, the *clpP* and *clpX* genes are separated by a 1.1 kb region containing the phosphotransferase *cicA* (Østerås et al., 1999; Fuchs et al., 2001), while *clpA* is co-transcribed with the conserved Clp protease adaptor *clpS* from a separate locus. A change in the ratio between ClpX and ClpA during σ^32^-dependent HSP induction could redirect ClpP proteolysis from ClpX-mediated functions in cell cycle regulation toward stress survival tasks mediated by ClpA (Østerås et al., 1999; Grünenfelder et al., 2004). In line with this idea, high ClpA abundance can inhibit ClpX function (Jenal, 1998), and ClpAP is competent to degrade unfolded proteins (Joshi and Chien, 2016; Liu et al., 2016). That ClpAP can degrade substrates primarily degraded by other proteases (Liu et al., 2016) has led to the suggestion that ClpAP functions as a compensatory protease. However, ClpAP performs specific tasks during optimal conditions as well, where it is the primary protease responsible for degrading the flagellar protein FliF and the division protein FtsA in the swarmer cell (Grünenfelder et al., 2004; Williams et al., 2014). The finding that ClpAP is able to curate abundances of other PQC network protein via degradation of the protease Lon (Barros et al., 2020) further indicates that regulation of ClpA, ClpX, and ClpP is complex.

The Lon protease regulates many points of *Caulobacter* cell cycle and development, including degradation of three essential cell cycle regulators; the methyltransferase CcrM (Wright et al., 1996), the swarmer cell-specific transcriptional regulator SciP (Gora et al., 2013), and the replication initiator DnaA (Jonas et al., 2013; Leslie et al., 2015). Lon-mediated degradation is in some cases regulated through its ability to bind DNA, as in how chromosomal DNA binding facilitates recognition and degradation of CcrM in the swarmer cell (Zhou et al., 2019). The ability of Lon to bind DNA, and the influence of DNA binding on its activity, may be particularly important in clearing damaged proteins from the chromosome during genotoxic stress (Zeinert et al., 2018). Through its ability to adjust the abundances of regulatory proteins and recognize the presence of unfolded proteins, Lon is ideally positioned to halt the cell cycle at the appearance of proteotoxic stress (Jonas et al., 2013; Leslie et al., 2015). The appearance of unfolded proteins has a dual effect on Lon degradation, firstly by increasing σ^32^-dependent expression of the protease, and secondly by stimulating Lon degradation of certain substrates (Jonas et al., 2013). The combined effects of this regulation provide a mechanism for halting the cell cycle during unfavorable conditions (Figure 2A), where upregulated and activated Lon degrades DnaA upon exposure to proteotoxic stress (Jonas et al., 2013). More recent work has suggested that Lon may be titrated from different substrate pools based on stress intensity, as mild temperature increases result in CcrM stabilization and increased expression of CcrM-regulated nucleotide metabolism genes that support rapid growth (Zeinert et al., 2020). In addition to roles in proteotoxic and genotoxic stress, Lon has additionally recently been found to be integrated into sensing and responding to low oxygen levels (Stein et al., 2020). How Lon activity and substrate selectivity can be targeted toward specific client protein pools in response to environmental changes remains an area of active research.

The contributions of other proteases to *Caulobacter* growth and survival are less well established, as in the case of the membrane-bound protease FtsH where the known substrate pool is limited to its conserved interaction with σ^32^ (Fischer et al., 2002). Curiously, a three amino acid deletion in the substrate recognition domain of the HslU chaperone subunit enables the HslUV protease to degrade σ^32^ in cells depleted of either DnaKJ or FtsH, where σ^32^ is normally stable (Schramm et al., 2017). This finding raises questions on if HslUV may perform redundant or degenerate roles with FtsH, however, the substrate pool and contribution of the HslUV protease to *Caulobacter* development and stress survival remains entirely uncharacterized. FtsH mutants exhibit growth and developmental defects during optimal conditions, and additionally are more sensitive to various stresses (Fischer et al., 2002), however, whether these phenotypes stem from the interaction of FtsH with σ^32^ or from degradation of other substrates remains to be discovered. In addition to the ATP-dependent proteases, *Caulobacter* possesses proteases specialized for degrading proteins in the membrane or periplasm, such as the membrane metalloprotease MmpA, which degrades the processed form of the polarity factor PodJ (Chen et al., 2006). Many more yet unidentified interactions between the PQC network and cell envelope proteins must be involved to coordinate stalk synthesis, divisome assembly, chemoreceptor placement, and the many other developmental events taking place across the membrane, with the requirements of optimal and stress conditions.

## Energy-Dependent Disaggregases Assist the PQC Network in Stress Survival

ClpB is a disaggregase that acts specifically to remediate aggregated protein, and consistent with its expression occurring exclusively during stress (Simão et al., 2005; Schramm et al., 2019), no phenotype is associated with its absence in optimal conditions. Deletion of ClpB results in an inability to dissolve stress-induced protein aggregates, and an associated reduction in the ability of *Caulobacter* to tolerate proteotoxic stress (Simão et al., 2005; Schramm et al., 2019). Protein aggregates are persistent in ClpB-deficient cells, and collaboration between DnaKJ/E and ClpB is the primary mechanism of resolving aggregated protein during sublethal heat stress in *Caulobacter*, although it is unknown if mistranslation-inducing antibiotic stresses, which do not induce HSP expression, depend as heavily on ClpB (Schramm et al., 2019). Persistent protein aggregates in *clpB* mutant cells have been used to study inheritance of insoluble protein deposits (Schramm et al., 2019), which has been hypothesized to underlie replicative decline in the stalked cell (Ackermann et al., 2003). While the majority of protein aggregates are swiftly dissolved when ClpB is functional, *Caulobacter* was found to share persistent aggregated protein deposits between stalked and swarmer cells (Schramm et al., 2019). The fidelity with which the PQC network curates the proteome as the stalked cell ages and experiences sequential stresses remains an open question.

ClpB is also solely responsible for mediating the shutoff phase of the σ^32^-dependent stress response in *Caulobacter* (Simão et al., 2005). This process is important for tolerating sublethal stress, where unfolding and aggregation of the stress-sensitive σ^73^ allows σ^32^ to interact with the RNAP instead (Simão et al., 2005). To ensure that σ^73^-regulated genes are not repressed indefinitely, ClpB reactivates σ^73^ from protein aggregates (Da Silva et al., 2003; Simão et al., 2005). This reactivation of σ^73^ restores its activity and allows it to compete with σ^32^ for association with the RNAP, which is a crucial step in recovering from or adapting to proteotoxic stress (Simão et al., 2005).

## Energy-Independent Holdases Assist the Major PQC Network Nodes in Surviving Specific Stresses

To cope with the demands of proteotoxic stress, *Caulobacter* also employs energy-independent holdases that collaborate with major nodes of the PQC network and assist in survival. Two small heat shock proteins, sHSP1 (CCNA_02341, referred to also as IbpA, but not to be confused with the inositol binding protein A of *Caulobacter*), and sHSP2 (CCNA_03706), are induced in response to protein unfolding, and may organize unfolded proteins in a disaggregation-ready state, as aggregates dissolve more slowly in their absence (Schramm et al., 2019). While sHSP accumulation is restricted to σ^32^-acting stresses, the sHSP1 protein has been confirmed as a ClpXP substrate, featuring the classical C-terminal AA degron (Bhat et al., 2013). It remains unresolved if the ClpXP protease might remove the highly expressed sHSP1 during later phases of stress recovery, when cells are returning to growth in optimal conditions, or effect its turnover during optimal conditions.

The chaperedoxin holdase CnoX (CCNA_00109) is both constitutively expressed and σ^32^-responsive, and participates in cellular redox homeostasis during optimal conditions by reducing disulfide bonds (Goemans et al., 2018a). Oxidative stress further activates CnoX, and it functions to protect approximately 90 proteins from aggregation until they can be transferred to DnaKJ and GroESL for refolding (Goemans et al., 2018a) (Figure 2B). Similarly to what is observed with sHSP1 and sHSP2, deletion of CnoX does not affect survival of proteotoxic stress (Goemans et al., 2018a; Schramm et al., 2019), however it is unresolved if other stress conditions *Caulobacter* frequently encounters might exhibit a higher requirement for these holdases. As CnoX interacts with cell cycle-regulated proteins (Goemans et al., 2018a, b), many open questions remain on the function of holdases during both *Caulobacter* development and stress. *Caulobacter* also possesses a homolog of trigger factor (CCNA_02042), which likely contributes to the *de novo* folding of cytoplasmic proteins, but remains so far unstudied. Furthermore, holdase regulation and collaboration in compartment-specific stress (Castanié-Cornet et al., 2014; De Geyter et al., 2016), such as regulation of the holdase SecB (CCNA_03858) in membrane protein transport, remains unaddressed in *Caulobacter*.

## Conclusion and Outlook

*Caulobacter* has become a powerful model to investigate how a prokaryotic PQC network integrates the demands of a developmental program with frequently encountered threats to proteostasis. Several mechanisms by which the highly conserved nodes of the PQC network ensure cell cycle progression during optimal conditions are known, and ongoing work has begun to reveal mechanistic insight into how these functions change when stress is encountered. Substrate trapping or depletion experiments connected to mass spectrometry have begun to characterize client proteins of the major chaperones and proteases (Bhat et al., 2013; Goemans et al., 2018a; Schroeder et al., 2020), and with this an understanding of how processes are regulated as individual proteins transit through the PQC network has become possible.

A theme emerging from recent work in *Caulobacter* is that the PQC network responds to a range of stress inputs and intensities with tailored and collaborative responses. For example, *Caulobacter* primarily dissolves aggregated protein deposits during stress recovery, except after high intensity stresses where dilution of aggregates in the growing population becomes the primary method of reducing insoluble protein (Schramm et al., 2019). Lon may also be directed toward different activities based on stress intensity; during mild temperature increases Lon has been linked to increasing dNTP pools to support DNA replication (Zeinert et al., 2020), whereas strong unfolding stress requires Lon-mediated destabilization of DnaA and prevention of DNA replication initiation until conditions improve (Jonas et al., 2013). Stress-responsive factors that tune the generalist PQC network to specific environmental conditions are also beginning to be identified, such as the chaperedoxin CnoX that responds to oxidative stress and collaborates with DnaKJ/E and GroESL (Goemans et al., 2018a). How PQC network functions change within the dynamic range of stress responses, and how nodes of the PQC network collaborate in effecting these responses is a developing area of *Caulobacter* PQC work.

Finally, work in *Caulobacter* is also beginning to address how the discrete proteomes of the swarmer and stalked cell interface with the PQC network. Cell cycle-restricted *Caulobacter* proteins that are particularly sensitive to aggregation have been described (Schramm et al., 2019), and as oscillations in transcriptome (Fang et al., 2013), proteome (Grünenfelder et al., 2001), and metabolome (Hartl et al., 2020) of *Caulobacter* during the cell cycle in optimal conditions have been identified, the field is open for investigating the interface between PQC machines and the dimorphic developmental program of *Caulobacter* during environmental changes.

## Author Contributions

Both authors contributed to the writing and final approval of the manuscript.

## Conflict of Interest

The authors declare that the research was conducted in the absence of any commercial or financial relationships that could be construed as a potential conflict of interest.
